# The impact of AI errors in a human-in-the-loop process

**DOI:** 10.1186/s41235-023-00529-3

**Published:** 2024-01-07

**Authors:** Ujué Agudo, Karlos G. Liberal, Miren Arrese, Helena Matute

**Affiliations:** 1 Bikolabs/Biko, Pamplona, Spain; 2 Departamento de Psicología, Universidad de Deusto, Avda. Universidad 24, 48007 Bilbao, Spain

**Keywords:** Human–computer interaction, Automation bias, AI, Decision-making, Human-in-the-loop, Compliance, Artificial intelligence

## Abstract

Automated decision-making is becoming increasingly common in the public sector. As a result, political institutions recommend the presence of humans in these decision-making processes as a safeguard against potentially erroneous or biased algorithmic decisions. However, the scientific literature on human-in-the-loop performance is not conclusive about the benefits and risks of such human presence, nor does it clarify which aspects of this human–computer interaction may influence the final decision. In two experiments, we simulate an automated decision-making process in which participants judge multiple defendants in relation to various crimes, and we manipulate the time in which participants receive support from a supposed automated system with Artificial Intelligence (before or after they make their judgments). Our results show that human judgment is affected when participants receive incorrect algorithmic support, particularly when they receive it before providing their own judgment, resulting in reduced accuracy. The data and materials for these experiments are freely available at the Open Science Framework: https://osf.io/b6p4z/ Experiment 2 was preregistered.

## Background

The presence of artificial intelligence algorithms and automated systems in public sector decisions (Araujo et al., 2020; Eubanks, 2018; O’Neil, 2016), such as social assistance (Civio, 2022; De-Arteaga et al., 2020; López-Ossorio et al., 2016), justice (Casacuberta & Guersenzvaig, 2018; Larson et al., 2016; Martínez-Garay, 2016; Niiler, 2019), health (Obermeyer et al., 2019; Raghu et al., 2019), and education (Alon-Barkat & Busuioc, 2022; Duncan et al., 2020), is becoming increasingly common.

Thus, many countries already use automated decision support systems which are often based on artificial intelligence (Solans et al., 2022). Examples are the United States (Berkman Klein Center, 2022), the United Kingdom (Ministry of Justice, 2013), China (Wei, 2019), Estonia (Niiler, 2019), Argentina (Ministerio Público Fiscal de la Ciudad Autónoma de Buenos Aires, 2020), Poland (Ministerstwo Sprawiedliwości, 2021), and Spain (Capdevila et al., 2015; Valdivia et al., 2022), in the judicial context, which is the focus of this article. In these cases, the system usually does not make the decision fully autonomously, but rather supports the human decision process (Araujo et al., 2020) in different ways, such as gathering and summarizing the information needed for the decision, or recommending a particular decision (Binns & Veale, 2021). This initiative of introducing humans into an automated decision process is known in the literature as a human-in-the-loop process. The idea is that this human presence should guarantee a better final decision due to human supervision of the system and appropriate intervention to prevent and/or mitigate errors that could be made by the automated system (Ponce, 2022; Portela & Álvarez, 2022).

Existing legislation and policy recommendations on automated decision systems (often designated by a variety of terms such as algorithm, artificial intelligence system, AI technology, or robot; European Commission, 2019) emphasize the right of citizens not to be subject to a fully automated decision, and point to the importance of the human presence in the process as a safeguard and protection against a possible erroneous or biased algorithmic decision (Green, 2022; Portela & Álvarez, 2022).

However, this approach is not without difficulties. Achieving appropriate interaction between humans and automated systems is complex because it requires, among other things, that the humans involved in the process have the skills, experience, motivation, and time to interpret and critically manage the information provided by the system (Ponce, 2022; Portela & Álvarez, 2022), can understand how these systems work, and are able to disagree with the automated system’s decision (Green, 2022) in the event of a conflict between human judgment and algorithmic recommendation (Valdivia et al., 2022).

There is a large body of empirical evidence questioning the human ability to disagree with or override an automated decision. In fact, for more than two decades, the scientific literature has pointed to a human tendency to use the information provided by support systems as a shortcut to avoid searching for or processing other relevant information. In this way, people demonstrate compliance with the system’s decision or delegate their decision to the system. Excessive human compliance when the system’s assessment is erroneous is often referred to in the engineering and artificial intelligence fields as *automation bias* (Cummings, 2004; Lyell & Coiera, 2017; Mosier & Manzey, 2019; Parasuraman & Mustapha, 1996), and this effect has been documented in domains as diverse as aviation, healthcare, military, and process control (see meta-analysis by Mosier & Manzey, 2019). An example of this automation bias is found in Lyell et al. (2017). In this study on drug prescribing using an automated decision support system, the researchers found that when the system erroneously indicated that a drug was not appropriate for a patient, prescribing errors increased by 56.9%.

In the judicial system, however, recent work reports a less consistent and less robust effect of this automation bias. On the one hand, there are cases of algorithm implementation that suggest excessive human compliance with system decisions, as in the case of RisCanvi, the system used for assessing the risk of recidivism of inmates in Catalonia, Spain. According to Saura and Aragó (2021), government officials using RisCanvi disagree with the algorithm only 3.2% of the time. This is so, even though, as shown in the most recent general report published on the performance of this system, RisCanvi has a positive predictive capacity of 18%, that is, only two inmates out of ten end up confirming the system’s prediction and reoffending after being classified as high risk (Capdevila et al., 2015; Martínez-Garay, 2016). However, this data about the poor predictive capacity of RisCanvi is not made visible when the system is used and is therefore likely to be unknown to the government officials who use it.

On the other hand, several empirical studies using similar forensic AI models, seem to suggest the opposite results (Green & Chen, 2019a, 2019b, 2021; Grgic-Hlaca et al., 2019; Portela et al., 2022; Skeem et al., 2020). For example, Grgic-Hlaca et al. (2019) conducted an experiment in which participants first had to predict, without AI support, whether some defendants would reoffend within two years. The researchers then showed the participants the recidivism prediction estimated by a computer program, and the participants were asked to indicate their prediction again. The researchers also showed the participants the accuracy rate of the computer program (68%). Only in a minority of cases did the participants adjust their prediction after seeing the computer’s estimate, showing a low level of automation bias. According to the authors, the 32% reported error of this system probably influenced the low bias observed. In addition, and as we will discuss below, the fact that the algorithmic prediction was presented after, rather than before, the participants provided their judgments, may have also been a critical factor in the low compliance observed in this study.

Moreover, in a related experiment, Green and Chen (2019a) manipulated the race of the defendant to assess how this data affected compliance with algorithmic support. They found that automation bias increased when the algorithm predicted a high risk of recidivism in cases where the defendant was black, and a low risk of recidivism in cases where the defendant was white. That is, participants agreed with the algorithm when it confirmed their own prejudices.

Quite possibly, these contradictions about the impact of automated system support on human-in-the-loop processes are due in part to the wide disparity in the methodological procedures used. Existing work on this topic evaluates the role of different human decision-makers performing different tasks, in different countries, in different domains, and with very different decision processes. Studies also vary in terms of whether or not participants are informed about the predictive accuracy of the algorithm; what the algorithm is called (decision support system, computer program, algorithm, or artificial intelligence among others); whether or not the system provides erroneous support; whether or not an explanation of the criteria followed by the system is provided; whether or not participants receive feedback on how accurate their decision was; and at what point participants receive support from the system, whether before or after making their own judgment.

Therefore, as we have already pointed out, we believe that the differences in the results obtained in the studies of automation bias in human-in-the-loop processes may be due in part to the variety of methodological procedures employed in these studies and in those models. Moreover, not all of those studies are a true reflection of the actual human decision-making processes used in the public sector, so they may not all have the same ecological value from an applied perspective. For example, in cases of actual implementation of automated decision systems in the public sector, system support is typically provided at the beginning of the decision process. Specifically, this process usually follows the following sequence (Chong et al., 2022; Solans et al., 2022): First, the system evaluates the available information and shows its assessment; then, the human is given just a few options: to validate, or to modify the system assessment. This sequence implies that human decision makers never explicitly emit their own judgments, but merely validate or modify the system’s assessments; and that the system support is received at the beginning of the process, establishing an order in the presentation of information that is likely to influence the processing of decision-relevant information by the human decision-maker (Marquardson & Grimes, 2018) and affect compliance and accuracy.

One example of this possible influence would be the anchoring bias (Rastogi et al., 2022). Anchoring bias is the tendency to over-rely on a piece of information we initially receive (the anchor) so that then we tend to adjust our final judgment based on that starting point or anchor (Epley & Gilovich, 2006; Tversky & Kahneman, 1974). In the case of human-in-the-loop processes, system support (e.g., suggesting a high, medium, or low risk of recidivism for an inmate) is presented before humans make their decisions. This human decision, as noted above, is usually limited to government officials confirming or modifying the assessment previously made by the system. Thus, this AI support could act as an anchor that conditions the human decision maker, whose final decision would be merely an adjustment to the system’s assessment.

Therefore, we conducted two experiments designed to test whether manipulating the time at which system support is presented in a human-in-the-loop process can help to increase the accuracy of the final decision, and reduce excessive compliance (i.e., automation bias) when the system makes errors. In order to recreate a decision process that is as close as possible to the real processes implemented in the public sector, our two experiments in the field of justice, simulated the RisCanvi system (Soler, 2013). As mentioned above, this system predicts the recidivism risk of inmates in Cataluña, Spain, and we use it merely as an example, because it includes the features common to the other systems described above: a specific sequence in the decision process (first system support, then validation or modification by the human decision-maker) and a very simple interactive interface consisting on just two buttons, one to validate and another one to modify the assessment of the system. Thus, in this type of human-in-the-loop process, human decision makers do not explicitly emit their own judgment. Instead, they only confirm or modify the assessment previously received from the system. We believe that this could probably favor human compliance with the AI assessment, which would act as an anchor for the human decision. As previously mentioned, people using RisCanvi agree with the algorithm 96.8% of the time (Saura & Aragó, 2021), even though the algorithm has only 18% positive predictive power (Capdevila et al., 2015; Martínez-Garay, 2016).

There are few studies of human-in-the-loop processes that have manipulated the time at which the system support is received, or that have presented algorithmic support at different points in the decision process (Buçinca et al., 2021; Echterhoff et al., 2022; Green & Chen, 2019b; Rastogi et al., 2022; Vicente & Matute, 2023) to study whether this support can cause an anchoring effect on the human decision or in any way affect the final decision. For example, Green and Chen (2019b) conducted an experiment in which their participants had to indicate, on a scale of 0 to 100, the probability that several inmates would fail to appear in court or would be arrested before trial. In one of the experimental conditions, the participants gave their judgment before the algorithmic assessment was shown. This assessment was sometimes incorrect, simulating the performance of real-world systems such as the COMPAS algorithm (Angwin et al., 2016). In this condition, in which the participants emitted their judgment before and after receiving the algorithmic support, the highest accuracy was obtained, as compared to other conditions in which the algorithmic assessment was provided before the participants’ judgment, or not displayed at all.

In another context, Buçinca et al. (2021) conducted an experiment in which participants had to identify the highest carbohydrate ingredient in a food dish in order to replace it with another dish with less carbohydrate but similar taste. They found that participants’ accuracy and compliance were affected by the moment at which they received erroneous support from an AI. The performance of participants who emitted their judgment before seeing the incorrect AI assessment was better than that of participants who saw the AI assessment first. In addition, the former group was also less compliant than the group who received the erroneous AI support before making the decision. Although none of the groups that received the incorrect AI support completely avoided the automation bias, the authors suggest that asking participants to emit their judgments before seeing the incorrect AI assessment may act as a cognitive forcing function. This cognitive forcing function would force users of decision support systems to think more analytically and disrupt the fast and heuristic reasoning that may lead them to show compliance (Lambe et al., 2016).

We believe that understanding the impact of human-AI interaction on automated decision processes can lead to more accurate decisions and less automation bias, because the current lack of conclusive evidence in this area is not slowing down the implementation of these automated decision support systems in the public sector, which is a concern. Therefore, as mentioned above, we conducted two experiments, inspired by real-world AI decision support systems such as RisCanvi. In these experiments, we manipulated the time at which algorithmic assessments are received in a human-in-the-loop process. Our purpose was to test whether this manipulation could contribute to improving collaborative human-AI decision-making by helping to reduce compliance when the system errs, and increase decision accuracy. That is, our purpose was not to test whether humans are more or less accurate than automated systems in making their assessments. Our aim was to evaluate the standard sequence of decision-making in human-in-the-loop processes, which, as noted above, consists of the system first showing its assessment, and then humans merely confirming or modifying that assessment, without at any time explicitly emitting their own judgment. We believe that such a sequence may favor an anchor bias that could affect the accuracy of decisions, even to the point of leading to excessive compliance when the system errs. If that were the case, we believe that changing the time at which the AI support is provided and, as suggested by Lambe et al. (2016), forcing the human to explicitly emit a judgment before receiving the system’s assessment, should be a good strategy to reduce the bias, and thus increase accuracy and reduce compliance.

## Experiment 1

This experiment simulates a human-in-the-loop process in which participants receive erroneous support from an AI system to decide the guilt of several defendants. Our purpose was to test whether forcing participants to explicitly make their judgment when they have not yet received the system’s erroneous assessment could improve the accuracy of the decision, as compared to when the biased AI support is the first step. Thus, we hypothesized that asking human decision-makers to emit their judgment before receiving the algorithmic assessment would improve the accuracy of their judgment and reduce their compliance with the AI incorrect support, that is, this should reduce their automation bias.

### Method

#### Participants

We recruited a sample of 150 participants (36.6% women, 62.7% men, 0.7% non-binary), aged 18 years or older (*M* = 33.2, *SD* = 11.4), through the Prolific Academic platform. Since our experiment, which was conducted online, was inspired by the automated decision system used in Spain, RisCanvi, we recruited a sample from this country. To do so, we used the “Nationality: Spain” and “First language: Spanish” filters on Prolific. Although to conduct an experiment as similar as possible to the real-world decision process, it would have been appropriate to use a sample of government officials linked to the penitentiary and judicial field, we opted for a sample of laypeople. This decision, in addition to facilitating recruitment, was supported by previous work on automated decision systems in justice, which states that the behavior of laypeople and professionals does not differ (Green & Chen, 2021).

The sensitivity analysis for the sample size showed that we had a power of 80% to detect small to medium-sized effects (*w* = 0.22). The online program randomly assigned each participant to one of two experimental groups: AIsupport→Judgment (*n* = 76), or Judgment→AIsupport (*n* = 74).

#### Design and procedure

After providing some basic demographic information (age and gender), all participants read the same instructions. The instructions told them that their task was to assess the probability that several defendants were guilty, based on witness testimonies. We also told them that they would count on the support of an Artificial Intelligence system. Next, we asked participants about their degree of confidence, both in their own ability and in the AI system, given that these two factors could affect the acceptance or rejection of algorithmic support, as noted by some researchers (Chong et al., 2022; Green & Chen, 2019a). Thus, participants had to indicate how confident they were that they would perform the task properly, and that the artificial intelligence system would adequately assess the defendants’ guilt. Then, the experiment proper begun.

The experiment consisted of three trials for each participant, and each trial consisted of three steps. Table 1 shows a summary of the three steps in each trial. In Step 0, the computer presented the participants with a criminal case to be judged and the testimonies associated to it. In order to use standardized materials, we used the criminal cases of the ForenPsy 1.0 normative bank of testimonies developed by Álvarez et al. (2023). This bank includes the description of three criminal cases (homicide, threats, and trespassing) with 15 testimonies each. In the study by Álvarez et al., the 45 testimonies were ranked by a sample of anonymous participants, who estimated the degree of innocence or guilt that each testimony suggested about each of three defendants.

**Table 1:** Design Summary of Experiments 1 and 2, Showing the Steps in each Trial

Group	Step 0	Step 1	Step 2
AIsupport→Judgment	Description of a criminal case and witness testimonies	AI support (Confirm or modify AI’s assessment)	Judgment (without AI Support being present)
Judgment→AIsupport		Judgment (without AI Support being present)	AI support (Confirm or modify AI’s assessment)

**Fig. 1:**
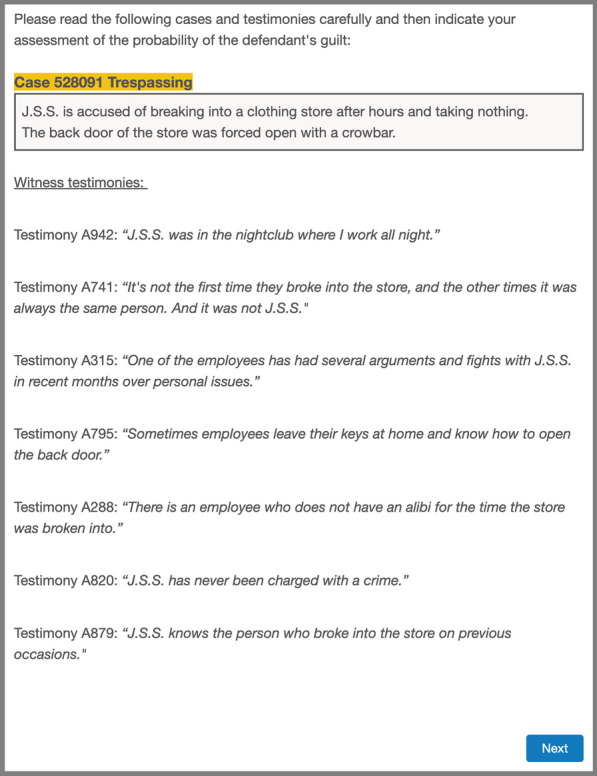
Example of a Screenshot of Step 0 (Description of a Criminal Case and Witness Testimonies), in Experiment 1 (Translated from Spanish). *Note*. In Experiment 1, each criminal case showed seven testimonies (of innocence in the example), while in Experiment 2, each case showed five testimonies

Thus, Step 0 in each trial consisted of a description of one of the three criminal cases of ForenPsy 1.0 (Álvarez et al., 2023), along with seven testimonies that suggested either innocence or guilt (see Fig. 1). Five of the seven testimonies clearly pointed to one of the verdicts (innocence or guilt), and the other two pointed in the same direction but were somewhat ambiguous, according to the ForenPsy calibration. The introduction of these two more ambiguous testimonies was intended to add realism to the trials. Some participants viewed the seven testimonies that suggested innocence, while some viewed the seven testimonies that indicated guilt. The type of testimonies that each participant received (i.e., innocence or guilt) was randomized. In addition, the order of presentation of each case and each testimony was randomized for each trial.

**Fig. 2:**
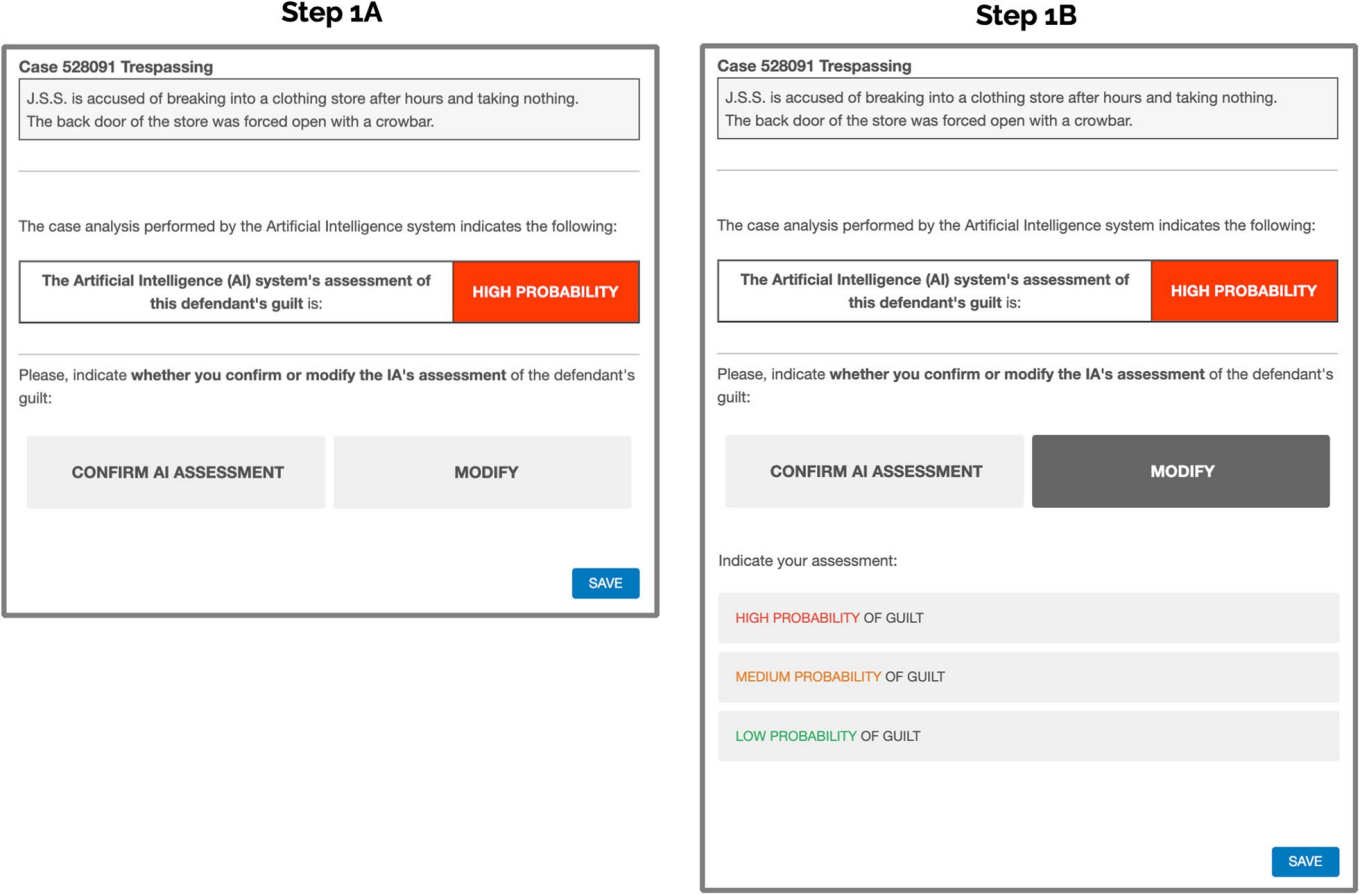
Step 1A shows a example of a Screenshot of Step 1 (Confirm or Modify AI’s Assessment) in the AIsupport→Judgment Group, in Experiments 1 and 2 (Translated from Spanish). *Note*. Step 1B was only shown to participants who clicked the Modify button. In that case, the change from 1A to 1B did not involve a screen change. The new information was displayed below the Confirm and Modify buttons on the same screen. The Judgment→AIsupport group viewed these screenshots in Step 2

Our main experimental manipulation took place in Steps 1 and 2 in each trial. The order in which these two steps occurred was reversed for each of two different groups (see Table 1). In the AIsupport→Judgment group, during Step 1, the participants were shown the probability of guilt estimated by a (fictitious) artificial intelligence system.^1^ The AI assessment of the defendant’s guilt that was shown to the participants could only take two values: high probability of guilt or low probability of guilt (see Fig. 2), so that it could be either congruent or contradictory with the verdict suggested by the testimonies presented during Step 0. In the first two trials, the system assessment was always correct, that is, it was congruent with the previously presented testimonies, which suggested either innocence or guilt according to the ForenPsy calibration. It was only in the last trial (the incorrect trial hereafter) that the system assessment was erroneous. In this trial the system always suggested the opposite verdict to that suggested by the testimonies. For example, if the testimonies presented had been rated in ForenPsy 1.0 as indicating innocence, the system suggested a high probability of guilt. And if the testimonies had been rated in ForenPsy 1.0 as suggesting guilt, then the system indicated a high probability of innocence. Thus, the accuracy of our fictitious system was 66% (one error out of three), a rate that we did not share with participants because government officers who use these systems usually do not receive this information either. This accuracy level is very similar to that reported by similar systems, such as RisCanvi (Capdevila et al., 2015), and COMPAS (Angwin et al., 2016).

On the same screen where the system’s assessment was shown, the participants of the AIsupport→Judgment group had to choose between confirming or modifying the assessment of the AI system, by clicking on the corresponding button. If participants chose to confirm the AI assessment, they proceeded directly to Step 2. If they chose to modify, a selectable list appeared below the button and the participants could modify the AI’s assessment by choosing one of these three options: high, medium, or low probability of guilt (see Fig. 2). The reason for our using a three-options response during Step 1, rather than using a more continuous and sensible scale, was because we wanted to use a measure as similar as possible to that commonly used by real life decision support systems implemented in the judicial system (i.e., high risk of recidivism, moderate and low). Moreover, we chose this more realistic three-point scale, instead of simplifying the scale and using only the two options that the AI assessment could show (high or low probability of guilt), in order to analyze whether, in the case that participants did not comply with the incorrect AI support, this implied that they were accurate in their decision (because their verdict was congruent with the one indicated by the testimonies) or that they did not know whether to follow the AI support or the verdict suggested by the testimonies, so they were not accurate (because they chose the medium probability of guilt). After modifying the AI assessment, these participants also proceeded to Step 2.

**Fig. 3:**
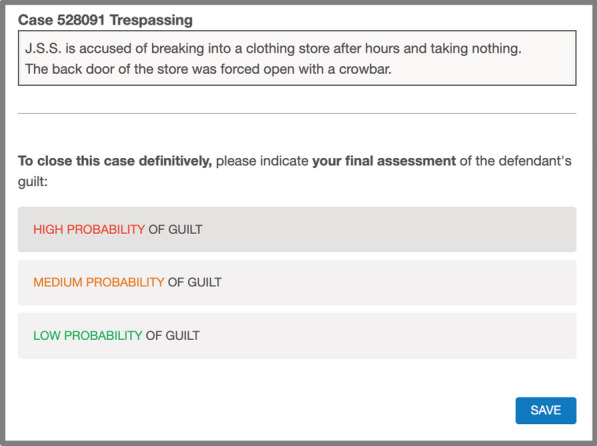
Example of Screenshots in Step 2 (Judgment Without AI Support) in the AIsupport→Judgment Group, in Experiment 1. *Note*. The Judgment→AIsupport group viewed this screenshot in Step 1. In that case, the phrase “To close this case definitively" did not appear on this screen, but on the corresponding Step 2 screen

In Step 2, the participants in the AIsupport→Judgment group were told that they had to indicate their final judgment on the defendant’s guilt. This final judgment was provided using a selectable list, that was identical to the one used when the participants chose to modify the AI’s assessment during Step 1 (i.e., high, medium, or low probability of guilt). This step may seem repetitive, but it was added in order to (a) obtain at least one personal judgment from all participants (i.e., even from those choosing just to confirm the AI assessment in the previous step), and (b) equate the number of times that the participants in both groups were asked to emit their judgment (see Table 1). That is, it was important that both groups had the identical type and number of tests so that only the time at which the AI assessment was shown would be a factor. Thus, if differences were observed when participants emitted their personal judgments (without the AI support being present) these differences could only be attributed to one group having already received the AI support in the previous phase. To make this request seem more natural, the Step 2 instructions informed participants in this group that the assessment they were to make was the one that definitively closed the case (see Fig. 3).

In the Judgment→AIsupport group, the only difference was that the order of steps 1 and 2 was reversed. That is, during Step 1, the participants in this group emitted their personal judgment about the probability of the defendant’s guilt without the support of the AI, and using the same three-points scale (high, medium, or low probability of guilt) as the other group. This was designed as a proposed improvement to the usual decision sequence of human-in-the-loop processes that do not explicitly ask humans to emit their judgment before receiving the AI assessment. We expected that forcing these participants to emit this judgment in a step prior to seeing the incorrect AI assessment might improve accuracy and reduce compliance in their final verdict. Next, in Step 2, the system assessment was shown and participants in this group had the opportunity to confirm or modify it, as did the participants of the other group during Step 1. If they decided to modify it, they were again shown the same three-point scale as in the previous step so that they could modify the AI assessment according to their criteria.

The absence of a real automated system and the use of the ForenPsy 1.0 set of testimonies allowed us to define and control in detail the appearance, format, and errors, of the supposed algorithmic support system. Thus, we controlled when the AI assessment was correct (the AI support was congruent with the testimonies), and when the AI assessment was incorrect (the suggestion of the AI support system was contrary to that of the testimonies).

Once participants completed the three steps for each of the three criminal cases that they received, all participants were asked again about their self-confidence and their trust in the system, using the same questions as those used at the beginning of the experiment. They were also asked about whether their job or studies were related to technology or the area of justice.^2^ When they finished, the participants were briefly informed about the real purpose of the study in a final debriefing stage.

### Results and discussion

#### Judgment accuracy without AI support present in the incorrect trial

We first analyze the accuracy when participants emit their personal judgment in the incorrect trial and the AI support is not present in that moment. It is important to keep in mind that the step in which the participants indicated their judgment without the AI support being present differed as a function of the group (see Table 1). While participants in the Judgment→AIsupport group assessed the defendants’ guilt on their own in Step 1, that is, before receiving the AI incorrect support in the next step, participants in the AIsupport→Judgment group did so in Step 2, that is, after having seen the AI assessment in Step 1. This allowed us to test whether judging a criminal case without having seen the incorrect AI assessment at any time, compared to having seen it in a previous step, resulted in a more accurate judgment.

**Fig. 4:**
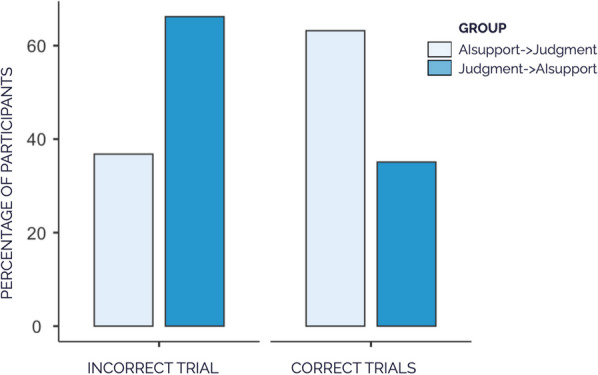
Percentage of Participants with Correct Assessments in Each Group by Type of Trial (Incorrect or Correct), in Experiment 1. *Note*. On the correct trials, the testimonies and the AI assessment that participants viewed in Steps 1 and 2 (according to the group) were congruent; on the incorrect trial, they were incongruent. Data of accuracy in the correct trials represent the percentage of participants whose judgments were accurate on both correct trials

As we expected, participants in the Judgment→AIsupport group (i.e., the group that judged the defendant without having seen the incorrect AI assessment) were more accurate in the incorrect trials than participants in the AIsupport→Judgment group. This can be seen in Fig. 4. A chi-squared test analyzing whether or not the participants were correct in their judgment confirmed that differences between groups were statistically significant, *χ*^*2*^ (1) = 12.95, *p* < 0.001, Cramer’s *V* = 0.29. Out of all participants in the Judgment→AIsupport group, 66.2% (49 out of 74) provided accurate judgments, compared to 36.8% of the participants in the AIsupport→Judgment group (28 of the 76) who showed accurate judgments. Thus, it appears that, as expected, emitting their personal judgment before seeing the incorrect AI assessment led to higher accuracy, as participants in the Judgment→AIsupport group were more accurate the participants in the AIsupport→Judgment group.

#### Judgment accuracy without AI support present in the correct trials

Next, we analyze accuracy when participants made their personal judgments (without AI support present at that time) in the correct trials, that is, the two trials in which the AI suggested the same verdict as the testimonies. Again, in these two trials, participants made their own personal judgments either in Step 1, that is, without having seen the correct AI assessment (Judgment→AIsupport group), or in Step 2, that is, after having seen the correct AI assessment in the previous step (AIsupport→Judgment group).

In order to perform this analysis, we classified participants as having been accurate in the correct trials if they made the correct assessments in both trials. Contrary to what happened in the incorrect trial, we found that participants in the AIsupport→Judgment group were generally more accurate than those in the Judgment→AIsupport group. According to the chi-squared, the association between accuracy and group was statistically significant, *χ*^2^ (1) = 11.8, *p* < 0.001, Cramer’s *V* = 0.28. In the AIsupport→Judgment group, 63.2% participants (48 of the 76 subjects) were accurate in both correct cases, while only 35.1% participants in the Judgment→AIsupport group (26 of the 74) were accurate in both cases (see Fig. 4). Thus, it appears that having received correct assessment from the AI at a step previous to emitting their personal judgment, as is the case in the AIsupport→Judgment group, lead the participants in this group to an increase in the accuracy of their judgment in the correct trials.

#### Compliance with AI assessment in the incorrect trial

Next, we analyze the compliance of participants (i.e., automation bias in this case) when they receive support from the AI and this assessment is incorrect. We classified participants as showing compliance in the incorrect trial if they clicked the button to confirm the AI’s assessment, or if, despite clicking the button to modify it, they finally selected the same probability of guilt as the AI had suggested (see Fig. 2). This could happen in different steps depending on the group: Step 1 in the AIsupport→Judgment group, and Step 2 in the Judgment→AIsupport group. We expected lower compliance from the participants in the Judgment→AIsupport group as compared to the other group because these participants had already judged the case by themselves in the previous step. Thus, we expected that their previous judgment would prevent the anchoring effect that may occur when the AI assessment was presented first, and could even serve, as suggested by Lambe et al. (2016), a cognitive forcing function. In sum, we would expect that this manipulation would make it easier for them to detect the error in the AI assessment and reduce the possible automation bias.

We constructed a contingency table to analyze the relationship between participants compliance in the incorrect trial and group, and found that only 25 of the 150 participants validated the erroneous assessment of the AI, thereby only 16.7% showed excessive compliance with the AI. Of these 25 participants, 10 belonged to the Judgment→AIsupport group and 15 to the AIsupport→Judgment group. This difference is so small that it does not allow further analysis of this effect of compliance.^3^

Next, we analyze whether this lack of compliance implies that participants were actually accurate in their judgments when confirming or modifying the incorrect AI assessment, and whether there is a difference in this accuracy between groups. Thus, we compared their performance during the step in which the AI support was present and they were simply asked to confirm or modify the AI assessment. That is, we compared Step 1 in the AIsupport→Judgment group against Step 2 in the Judgment→AIsupport group. This allows us to test whether our proposal to force explicit judgment at the beginning of the process (which occurs in the Judgment→AIsupport group) improves the accuracy of the standard sequence of human-in-the-loop processes that do not ask for explicit human judgment before the AI support is presented (as mimicked in the AIsupport→Judgment group). We are interested in this comparison rather than comparing the final decision between groups at Step 2, because our proposal is not to introduce explicit judgment at any point in the process, but to force it at the beginning, as opposed to the usual practice of presenting AI support at the beginning.

According to the chi-squared, the association between accuracy and group was not statistically significant, *χ*^2^ (1) = 1.29, *p* = 0.256, Cramer’s *V* = 0.09. In the AIsupport→Judgment group, 34.2% participants (26 of the 76) were accurate in their decision, while 43.2% participants (32 of the 74) were accurate in the Judgment→AIsupport group. It appears that although forcing judgment at the beginning of the process in the Judgment→AIsupport group produces more accurate decisions when the incorrect AI assessment has not been seen, receiving this incorrect support in the next step impairs participants’ final verdict by reducing their accuracy and aligning it with the levels of the AIsupport→Judgment group.

It should be noted that in order to simulate a real-life human-in-the-loop decision process in our experiment, we used a three-level scale to request the participants assessments of guilt in a way similar to that used by AI decision support algorithms in the judicial systems. We consider the contribution of this experiment to be valuable precisely because we have attempted to simulate a real decision process with AI-human interaction. However, we were aware that such a procedural decision implied choosing a scale with low sensitivity, so we decided to use a more sensible 0–100 scale in our next experiment. Thus, we conducted a new experiment in which we modified some of the previous procedural decisions, seeking greater robustness in the results at the cost of a small reduction in the ecological value of the experiment.

## Experiment 2

**Fig. 5:**
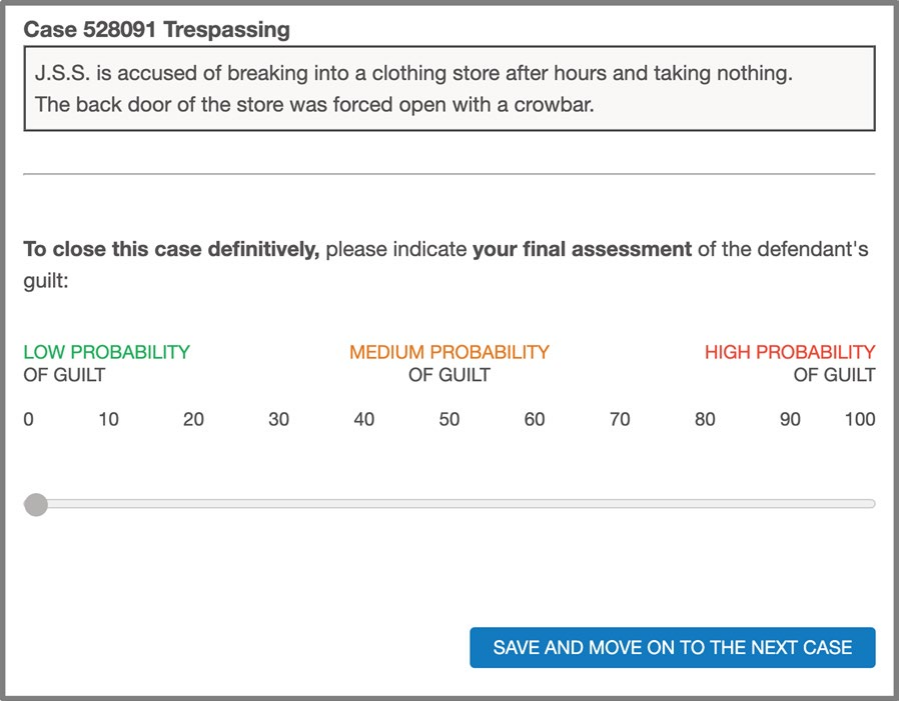
Example of a Screenshot in Step 2 (Judgment Without AI Support Present) in the AIsupport→Judgment Group, in Experiment 2. *Note*. To indicate when the steps of each case were completed, in this Experiment 2 we changed the literal of the "Save" button from Experiment 1 to "Save and move on to the next case". The Judgment→AIsupport group viewed this screenshot in Step 1. In that case, the phrase “To close this case definitively” of the instructions did not appear on this screen, but on the Step 2 screen

The aim of this experiment is to replicate the results of Experiment 1, and to obtain more robust results and generalize them to a larger sample. To this end, we made three main modifications to the previous experiment. First, we changed the scale on which participants made their assessments from the three-point scale used in Experiment 1 (simulating a real-world AI decision support systems) to a more standard 0 to 100 scale used in psychological research (see Fig. 5). This change allows for more sensible measurements and also facilitates the use of more robust statistical analyses.

Second, we increased to nine (instead of three) the number of criminal cases to be judged so that we could also increase sensitivity in this way. This will also allow us to compare the participants’ judgments in three incorrect cases rather than just one incorrect case, and in six correct cases rather than two. The accuracy ratio is maintained at a real-world level of 66%. In addition, we also eliminated the ambiguous testimonies from the cases presented and used only the five testimonies of each case that pointed most clearly to either innocence or guilt. We decided to eliminate ambiguity because if even when the materials are very easy and the verdict is obvious, participants would be misled by the erroneous AI support, then we would have clear evidence of a serious problem, with people following AI errors even in cases that they could easily resolve on their own.

Finally, we expanded the sample of participants not only in number (260 participants in this experiment) but also in diversity: although we maintained Spanish as the language of the study, we opened participation to people from any country. This experiment was preregistered in https://aspredicted.org/ph9br.pdf.

As in Experiment 1, we expected that participants who receive the erroneous AI support at the beginning of the process (group AIsupport→Judgment) would show more compliance and lower accuracy than those who emit their own judgment before receiving the incorrect AI support (group Judgment→AIsupport). In addition, we expected higher accuracy in the judgments of participants in the Judgment→AIsupport group on incorrect trials as compared to the judgments of the AIsupport→Judgment group.

### Method

#### Participants

We recruited a sample of 260 participants (42.3% women, 54.6% men, 3.1% non-binary), aged 18 years or older (*M* = 30.7, *SD* = 9.23), through the Prolific Academic platform. We used Prolific’s internal selection service to recruit this specific sample: participants over the age of 18 who had not previously participated in other experiments conducted by our research team on the Prolific platform, with Spanish as their first language, but from any country. Thus, the most represented nationalities were Mexican (40% of participants), Spanish (37.7%), and Chilean (8.5%), but there were also participants from Italy, USA, Venezuela, Peru, and Colombia, among others.

The sensitivity analysis for the sample size showed that we had a power of 80% to detect small effects (*d* = 0.10). As in the previous experiment, participants were randomly assigned to one of two experimental groups: AIsupport→Judgment (*n* = 132), or Judgment→AIsupport (*n* = 128).

#### Design and procedure

The design and procedure were very similar to those of the previous experiment. First of all, participants read the instructions and provided their age and gender. This time we did not ask them about their confidence in their own abilities and in the system’s abilities, neither at the beginning nor at the end of the study, because these measures did not affect the results in Experiment 1.

Then we presented the cases to be judged, with some changes from the previous experiment which we describe next. This time, each participant viewed nine trials (nine criminal cases) instead of three. In order to use the ForenPsy 1.0 standardized materials already developed and tested by Álvarez et al. (2023), we had used in Experiment 1 only three criminal cases (two correct cases and one incorrect case). However, to gain sensitivity in Experiment 2, we decided to increase the number of cases to nine. These nine trials were presented grouped by offense type (three for homicide, three for threatening, and three for trespassing), with both these offense groupings and the trials within these groupings presented in random order. Of the nine trials, three were the same cases used in Experiment 1, based on the ForenPsy 1.0 standardized set (Álvarez et al., 2023). The other six trials were created using the ChatGPT4 AI large language model (OpenAI, 2023), which we edited for clarity and consistency.

Thus, in each of the nine trials, Step 0 consisted of a cover story describing a criminal case and five testimonies that clearly indicated either an innocent or guilty verdict for the defendant. To make the verdict more obvious to participants and thus to better control when the AI assessment would be incorrect, we eliminated the two ambiguous testimonies per case that we had used in Experiment 1, thus our using only five testimonies per case in this experiment. In order to weight the testimonies of the six new cases created for this experiment, we conducted a previous study with a sample of 52 volunteers^4^ to calibrate and select the testimonies that most clearly indicated a verdict of innocence or guilt.

Like in Experiment 1, the proportion of cases with erroneous AI support was 33%, that is, three of the nine cases (one of homicide, one of threats, and one of trespassing) in this experiment were incorrect. Although the order in which the trials for each crime were presented was randomized, we forced the first trial that each participant saw to never be an incorrect trial in order to build some confidence in the AI system.

The experimental manipulation took place in Steps 1 and 2. In the AIsupport→Judgment group, in Step 1, participants viewed the AI system’s assessment (low or high probability of guilt, as in the Experiment 1). In the incorrect cases, the AI assessment was incongruent with the previously presented testimonies and suggested the opposite verdict. As in Experiment 1, participants were asked on the same screen whether they wanted to confirm the AI assessment or modify it. If they decided to modify it, they could use the same three-point scale as in the previous experiment (high, medium, or low probability of guilt). Next, in Step 2, participants were asked to indicate their final assessment. In this experiment, this judgment was indicated on a more standard 0–100 judgment scale (where 0 was low probability of guilt, and 100 was high probability of guilt; see Fig. 5).

In the Judgment→AIsupport group, the order of Steps 1 and 2 was reversed, with participants emitting their judgment on the defendant’s probability of guilt on the 0–100 points scale in Step 1, and then viewing the AI assessment and confirming or modifying it as their final verdict in Step 2. Finally, all participants indicated whether their work or studies were related to technology or justice, and were debriefed and informed about the true purpose of the study.

### Results and discussion

#### Judgment accuracy without AI support present in the incorrect trials

We first analyze the difference in judgments between groups when participants judged the defendants in the incorrect trials (three incorrect cases, one for each of the three types of crimes). Since we used a 0–100 scale to measure the participants judgments in this experiment, we focused on the difference in judgments between groups to analyze accuracy.

**Fig. 6:**
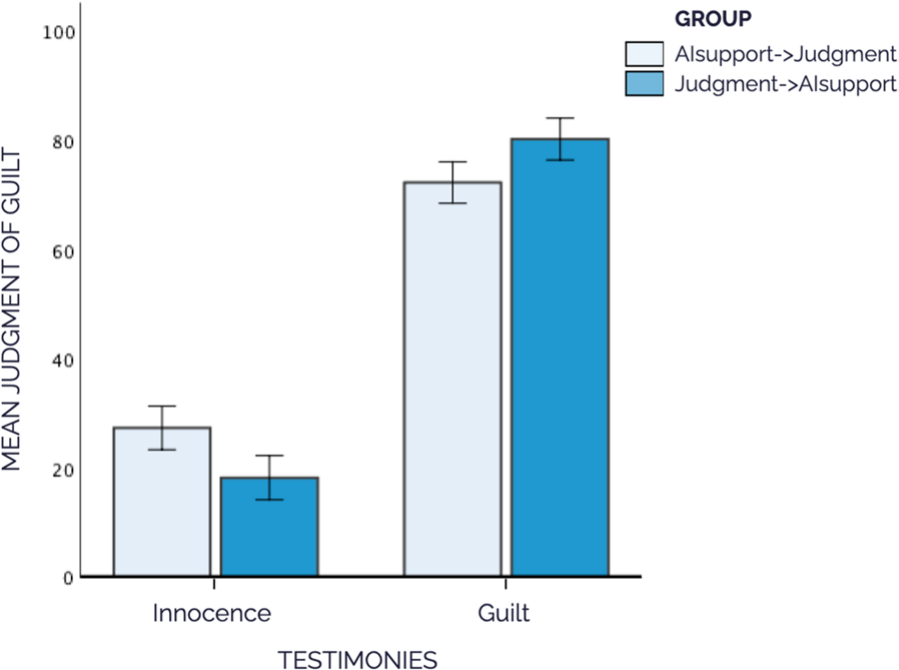
Mean Judgment of Guilt in Incorrect Trials, by Type of Testimonies and Group, in Experiment 2. *Note*. Error bars 95% CI

Figure 6 summarizes the results. As can be seen in this figure, the mean accuracy of judgments in the Judgment→AIsupport group is higher than in the AIsupport→Judgment group, regardless of whether the participants viewed testimonies pointing to innocence or guilt. These impressions were confirmed by a 2 (testimonies: innocence, guilt) × 2 (group: AIsupport→Judgment, Judgment→AIsupport) mixed ANOVA with mean judgments in the incorrect trials as the dependent variable.^5^ This ANOVA showed a main effect of testimonies, *F*(1, 188) = 636.4, *p* < 0.001, *η*^*2*^_*p*_ = 0.772; and no main effect for group, *F*(1, 188) = 0.104, *p* = 0.747, *η*^*2*^_*p*_ = 0.001. However, and as we expected, we observed a Testimonies x Group interaction, *F*(1, 188) = 16.3, *p* < 0.001, *η*^*2*^_*p*_ = 0.080.

Subsequent post-hoc comparisons (Tukey correction) showed that, as we expected, participants in the Judgment→AIsupport group judged defendants in the incorrect cases as less guilty when the testimonies indicated innocence (*M* = 18.1, *SD* = 15.2, *t*(369) = -3.26, *p* = 0.007, *d* = -0.53) than did participants in the AIsupport→Judgment group (*M* = 28.2, *SD* = 22.5). In addition, when the testimonies indicated guilt, the Judgment→AIsupport group judged the defendants in the incorrect cases to be more guilty (*M* = 79.8, *SD* = 15.7, *t*(369) = 2.83, *p* = 0.025, *d* = 0.48) than the AIsupport→Judgment group (*M* = 70.8, *SD* = 21.0). These results indicate that participants in the Judgment→AIsupport group were more accurate in their judgments. Thus, it seems that those participants who emitted their judgments after having received the incorrect AI support were negatively influenced by it, and their accuracy was reduced.

#### Judgment accuracy without AI support present in the correct trials

Next, we analyzed the participants’ judgments in the correct trials (six of the nine cases used in this experiment). In Experiment 1, we had found an opposite result in the correct trials as compared to the incorrect trial: participants in the AIsupport→Judgment group were more accurate in these trials than those in the Judgment→AIsupport group. Because we changed the judgment scale in Experiment 2 in order to have a more sensitive measure, we now were able to conduct a 2 (testimonies: innocence, guilt) × 2 (group: AIsupport→Judgment, Judgment→AIsupport) mixed ANOVA with the mean judgments in the correct trials as the dependent variable. We found a main effect of testimonies, *F*(1, 249) = 2453.4, *p* < 0.001, *η*^2^_*p*_ = . 908); no main effect of group, *F*(1, 249) = 0.43, *p* = 0.515, *η*^2^_*p*_ = 0.002; and a Testimonies x Group interaction, *F*(1, 249) = 5.28, *p* = 0.022, *η*^2^_*p*_ = 0.021. In order to look for potential between-group differences when the testimonies indicated innocence and when they indicated guilt, we conducted post-hoc comparisons, with Tukey correction. These showed no statistically significant differences between groups when the testimonies indicated innocence (*t*(491) = 1.28, *p* = 0.575, *d* = 0.16; Judgment→AIsupport group, *M* = 18.9, *SD* = 12.8; AIsupport→Judgment group, *M* = 16.9, *SD* = 11.5), nor when the testimonies indicated guilt (*t*(491) = −2.15, *p* = 0.139, *d* = −0.23; Judgment→AIsupport group, *M* = 77.8, *SD* = 16.1; AIsupport→Judgment group, *M* = 81.3, *SD* = 13.8). Thus, we found no differences between groups when participants made their judgments without the AI support being present on the correct trials. It seems that the between group difference observed in the correct trials of Experiment 1 disappears when more cases and a more standardized and sensitive scale is used in this experiment.

#### Compliance with AI assessment in the incorrect trials

We also analyzed the compliance of the participants with the incorrect AI assessment. We classified participants as compliant with the AI assessment if they showed compliance in at least one of the three incorrect trials. We expected the Judgment→AIsupport group to be less compliant than the AIsupport→Judgment group. Although we had not found this difference between groups in Experiment 1 due to the low compliance rate in both groups in that study, we expected to observe this result in Experiment 2, as we increased the sample size and the number of incorrect trials.

We conducted a chi-square test to analyze the difference between groups in participants’ compliance. We did not find a statistically significant difference in compliance between groups, *χ*^2^ (1) = 0.37, *p* = 0.545, Cramer’s *V* = 0.04. In the AIsupport→Judgment, 24.2% of participants showed compliance on at least one of the three incorrect trials in which the AI assessment was incorrect (32 participants out of 132); a very similar percentage was observed in the Judgment→AIsupport group, where 21.1% of the participants showed compliance (27 participants out of 128).

Finally, although we found no statistical differences between the groups in compliance, we must point out that the erroneous AI support again negatively affected decisions accuracy in this experiment. We compared the percentage of participants who were accurate on the three cases when they received the incorrect AI assessment (at Step 1 in the AIsupport→Judgment group and at Step 2 in the Judgment→AIsupport group), and we found that only 29.5% of participants in the AIsupport→Judgment group (39 out of 132) and 31.3% of participants in the Judgment→AIsupport group (40 out of 128) were accurate. There were no significant differences in accuracy between groups in their AIassisted step, *χ*^2^ (1) = 0.09, *p* = 0.765, Cramer’s *V* = 0.02. Again, even though participants in the Judgment→AIsupport group emitted more accurate judgments than the other group during the step in which the AI support was absent (which for them took place at the beginning of the task), when they received incorrect AI support, the accuracy of their decisions was impaired to the level of that of the other group.

## General discussion

Even though there is no clear consensus on whether automated decision systems with human-in-the-loop processes contribute to better decision-making, their use is increasing in many different fields, including the public sector. The present research was designed to better understand how an automated decision system can impact decisions in the legal context. Our experiments suggest that human judgment can be affected when AI support is received.

In cases in which the AI assessment is incorrect, it seems that the human verdict will be more accurate if it is emitted before receiving the erroneous AI support. In both Experiments1 and 2, we found that when participants emitted their judgment before receiving the incorrect AI support (Step 1 in the Judgment→AIsupport group), their judgment was closer to the one indicated by the testimonies than when participants emitted their judgment after receiving erroneous AI support (Step 2 in the AIsupport→Judgment group).

In cases in which the AI assessment is correct, we found that although receiving the correct AI support in a previous step appeared to make the judgments in the AIsupport→Judgment group more accurate than those in the Judgment→AIsupport group in Experiment 1, this difference was not statistically significant in Experiment 2, which included a larger and more heterogeneous sample, a larger number of trials, and a more sensitive scale to measure the judgments than Experiment 1. This suggests that correct AI support in a human-in-the-loop process is not as beneficial as it may seem, whereas incorrect AI support is critical because it increases human error. Thus, our experiments show a possible anchoring effect of incorrect AI support on the human decision. Receiving the incorrect support at the beginning of the process impaired the subsequent explicit judgment of participants in the AIsupport→Judgment group.

It should also be noted that we did not find excessive compliance of participants with the erroneous AI support in either experiment. It is possible that participants were aware of the incongruence between the verdict indicated by the testimonies and the incorrect assessment of the AI, which prevented them from automation bias. This result, which is in the line with more recent work on this topic (De-Arteaga et al., 2020; Grgic-Hlaca et al., 2019; Portela et al., 2022), contrasts with the high compliance that some automated systems, such as RisCanvi, show outside the laboratory (above 95%; Saura & Aragó, 2021. Future research efforts are necessary to understand such discrepancies and their impact. Importantly, this lack of compliance did not imply a more accurate decision. In both groups, in the step where they had to confirm or modify the incorrect assessment of the AI, the accuracy rate was low, even for the group that had previously been forced to emit their judgment and had done so accurately (i.e., Judgment→AIsupport group). These results suggest that while it may seem advisable for people involved in human-in-the-loop processes to explicitly report their judgments at the beginning, rather than merely supervising the AI and confirming or modifying the AI’s assessments, AI errors are very likely to compromise their final decision even when those AI errors occur after accurate human judgment. Therefore, it is probably better that the human judgment occurs first, and then the AI, rather than the human, provides a second opinion, in order to detect (and warn) of potential human errors. But then again, we will still need a third party (an external human auditor, or a human committee) that should have the last word and be able to critically analyze any potential discrepancies in this human-AI collaboration.

It is important to note that our experiments aimed to simulate the real-world systems, such as RisCanvi, in order to recreate the standard process of this type of automated systems. They were not experiments to evaluate RisCanvi. In fact, RisCanvi has more human intervention in the decision process (e.g., a human is required when selecting the information to be considered or not by the system, and when a government official decides to modify the risk estimated by the system, which requires final validation by a different person; Portela & Álvarez, 2022), than other well-known automated decision systems (such as COMPAS or the automated border control system of Frontex; Portela & Álvarez, 2022). These differences in the amount and purpose of human intervention in the various existing automated decision systems can affect the accuracy of the decisions making when using those systems, as well as human compliance with the system’s support.

In our experiments, we probably might have been able to get higher levels of compliance from participants by, for example, introducing more ambiguous testimonies into the cases so that the verdict in those cases would be less obvious. However, our goal was not to achieve a high level of compliance. We decided to eliminate ambiguity because if even when the materials are very easy and the verdict is obvious, participants would be misled by the erroneous AI support, then we would have clear evidence of a serious problem, with people following AI errors even in cases that they could easily resolve on their own.

The present experiments show that certain details in the interaction between humans and AI, such as the timing of the presented AI assessment, and whether or not humans are asked to emit their judgments explicitly, can have an important impact on decisions. Interestingly, these are details that are not usually considered when the convenience of implementing these systems is evaluated, or when these algorithms are audited, internally or externally, since in these cases the focus is usually placed on the technical aspects of the performance of the algorithm itself and not on its interaction with the people who use it (Buçinca et al., 2021; Green, 2022). Our research highlights the need to consider not only the technical aspects, but, most importantly, the human-AI interaction when evaluating or auditing these systems, because aspects such as the time when government officials receive the IA assessment, whether or not they make their judgment before seeing the AI assessment, or whether or not they are aware of, for example, the error rate of the system, can have a very large impact on the decisions made with human-in-the-loop processes. Indeed, those aspects would determine whether the AI support primes human decisions in the direction valued by the AI (when the AI acts first), or whether it is just left the role of providing a second opinion after the human has already emitted a judgment. Our experiments also suggest that it is important for the humans involved in these processes to have the skills, experience, and time to interpret and manage the information provided by the system (Ponce, 2022; Portela & Álvarez, 2022), to be informed about the error rate of the systems they supervise, and to have the ability (and the incentives) to be critical and to disagree with system decisions when necessary. As has already been shown, the influence of algorithmic support and recommendation on human decisions, both public and private, is often underestimated (Agudo & Matute, 2021).

Although in this research we have used accuracy as a measure to assess the human-in-the-loop process, decisions in the field of justice must be fair, correctable, and ethical among other things, in addition to being accurate (Green & Chen, 2019b). Thus, all of these aspects need to be considered as well, and not just the predictive accuracy of the system, when determining how beneficial it would be to deploy automated decision systems in the public domain. In this regard, we believe that a critical analysis of the convenience of establishing human-in-the-loop processes is necessary; not because we believe that it is better to make decisions in a fully automated way, but because we consider that the role of the human in an automated process hides certain pitfalls that need to be investigated in detail.

First, although human oversight is proposed as a safeguard in high-risk automated decisions (European Commission, 2019), there are critical issues such as those mentioned above, related to the experience of these humans, the time they have available, the responsibility and agency they have, their motivation and incentives, etc., that can turn human-in-the-loop processes into “quasi-automate” processes where the human contributes almost nothing (Wagner, 2019) and even provides a false sense of security (Ponce, 2022), as our results suggest. Indeed, it is worth noting that when the systems are accurate, their success is usually celebrated, emphasizing the crucial role of the algorithm in that accuracy; when they err, however, it is humans who are blamed for their lack of oversight or their automation bias.

Importantly, it has been noted that, it is more difficult for humans to supervise and judge the accuracy of an algorithm’s prediction than to make their own assessments and predictions (Green, 2022). Thus, as noted above, it is possible that rather than having humans supervising AI decisions, a better strategy could be to let humans make the decisions, while using AI tools to provide a second opinion and to alert humans of possible human error that they may detect; and then having a human auditor or a human committee analyzing any potential human-AI discrepancies.

Our results contribute to an increasing amount of scientific evidence suggesting that, before implementing these systems, it is necessary to consider which decisions it makes sense to automate and how to do it. In so doing, it should be taken into account, for example, whether it is a decision where the accuracy of the automated systems is far superior to human accuracy; or whether, on the contrary, it is a decision where a wide variety of criteria must be taken into account (as we have mentioned, in the case of judicial decisions, which must be fair, correctable and ethical, in addition to being accurate), and therefore it is not appropriate to use automated systems. Not all decisions are suitable for automation, nor does it seem desirable for us as humans to rely on decision-making processes in which the person involved in those processes is a clear candidate to be signaled out as the error of the system.

## Data Availability

The data and materials for this experiment are freely available at the Open Science Framework: https://osf.io/b6p4z/. Experiment 2 was preregistered in https://aspredicted.org/ph9br.pdf.

## Footnotes

1 Although automated decision-making in justice is used both to decide about past events (e.g., the conviction of a defendant) and to estimate the probability of future events (e.g., the aforementioned risk of recidivism of a defendant), we opted for the first option in our experiment as we considered that it would be easier for a non-expert in justice participant to decide on a past action than to predict a future action.

2 Of the total number of participants, only a few reported work or educational experience in justice (*n* = 12) or technology (*n* = 35).

3 In addition, these low compliance data did not allow us to analyze whether participants’ self-confidence or confidence in the system might contribute to increased automation bias.

4 The sample of 52 participants (69.2% men, 28.8% women; 2% no response) were employees of the same company (a Spanish technology consulting company) and over 18 years old (*M* = 35.6, *SD* = 8.92).

5 Each participant's three incorrect trials were randomly presented with some of them suggesting innocence and some suggesting guilt. This randomness implies that some participants received testimonies of innocence in the three incorrect cases, some received testimonies of guilt in all three cases, and some received some cases with innocence testimonies and some with guilt testimonies. This is reflected in the ANOVA degrees of freedom. In the AIsupport→Judgment group, the total number of trials with testimonies of innocence presented to participants was *n* = 117 and the number of trials with testimonies of guilt was *n* = 111; in the Judgment→AIsupport group, the number of trials with testimonies of innocence presented to participants was *n* = 112 and the number of trials with testimonies suggesting guilt was *n* = 110.

## References

[CR1] Agudo U, Matute H (2021). The influence of algorithms on political and dating decisions. PLoS ONE.

[CR2] Alon-Barkat S, Busuioc M (2022). Human-AI interactions in public sector decision-making: ‘Automation Bias’ and ‘Selective Adherence’ to algorithmic advice. Journal of Public Administration Research and Theory.

[CR3] Álvarez, M., Martínez, N., Agudo, U., & Matute, H. (2023). *ForenPsy 1.0.* Retrieved from https://osf.io/detn4/

[CR4] Angwin, J., Larson, J., Mattu, S., & Kirchner, L. (2016). Machine bias: There’s software used across the country to predict future criminals. And it’s biased against blacks. *ProPublica*. https://www.propublica.org/article/machine-bias-risk-assessments-in-criminal-sentencing

[CR5] Araujo T, Helberger N, Kruikemeier S, de Vreese CH, de Vreese CH (2020). In AI we trust? Perceptions about automated decision-making by artificial intelligence. AI & Society.

[CR6] Berkman Klein Center. (2022). *Risk assessment tool database*. Berkman Klein Center. https://criminaljustice.tooltrack.org/

[CR7] Binns R, Veale M (2021). Is that your final decision? Multi-stage profiling, selective effects, and Article 22 of the GDPR. International Data Privacy Law.

[CR8] Buçinca Z, Malaya MB, Gajos KZ (2021). To trust or to think: Cognitive forcing functions can reduce overreliance on AI in AI-assisted decision-making. Proceedings of the ACM on Human-Computer Interaction.

[CR9] Capdevila, M., Blanch, M., Ferrer, M., Pueyo, A., Framis, B., Comas, N., Garrigós, A., Boldú, A., Batlle, A., & Mora, J. (2015) *Tasa de reincidencia penitenciaria 2014*. Centre d’Estudis Jurídics y Formació Especialitzada de la Generalitat de Catalunya. https://cejfe.gencat.cat/web/.content/home/recerca/cataleg/crono/2015/taxa_reincidencia_2014/tasa_reincidencia_2014_cast.pdf

[CR10] Casacuberta, D., & Guersenzvaig, A. (2018). Using Dreyfus’ legacy to understand justice in algorithm-based processes. *AI & Society*, 1–7. 10.1007/s00146-018-0803-2

[CR11] Chong L, Zhang G, Goucher-Lambert K, Kotovsky K, Cagan J (2022). Human confidence in artificial intelligence and in themselves: The evolution and impact of confidence on adoption of AI advice. Computers in Human Behavior.

[CR12] Civio. (2022). *La Justicia impide la apertura del código fuente de la aplicación que concede el bono social*. https://civio.es/novedades/2022/02/10/la-justicia-impide-la-apertura-del-codigo-fuente-de-la-aplicacion-que-concede-el-bono-social/

[CR13] Cummings, M. (2004). Automation bias in intelligent time critical decision support systems. In *AIAA 1st Intelligent systems technical conference. american institute of aeronautics and astronautics*. 10.2514/6.2004-6313

[CR14] De-Arteaga, M., Fogliato, R., & Chouldechova, A. (2020). A case for humans-in-the-loop: Decisions in the presence of erroneous algorithmic scores. In *2020 CHI Conference on human factors in computing systems*, 1–12. 10.1145/3313831

[CR15] Duncan, P., McIntyre, N., & Levett, C. (2020). Who won and who lost: when A-levels meet the algorithm. *The Guardian*. https://www.theguardian.com/education/2020/aug/13/who-won-and-who-lost-when-a-levels-meet-the-algorithm

[CR16] Echterhoff, J. M., Yarmand, M., & McAuley, J. (2022). AI-moderated decision-making: Capturing and balancing anchoring bias in sequential decision tasks. In *Proceedings of the 2022 CHI conference on human factors in computing systems (CHI '22), 161,* 1–9. 10.1145/3491102.3517443

[CR17] Epley N, Gilovich T (2006). The anchoring-and-adjustment heuristic: Why the adjustments are insufficient. Psychological Science.

[CR18] Eubanks, V. (2018). *Automating inequality: How high-tech tools profile, police, and punish the poor*. St. Martin’s Press.

[CR19] European Commission. (2019). *Ethics guidelines for trustworthy AI*. https://ec.europa.eu/digital-single-market/en/news/ethics-guidelines-trustworthy-ai

[CR20] Green, B. (2022). The flaws of policies requiring human oversight of government algorithms. *Computer Law and Security Review, 45*. 10.1016/j.clsr.2022.105681

[CR21] Green, B., & Chen, Y. (2019a). Disparate interactions: An algorithm-in-the-loop analysis of fairness in risk assessments. In *Conference on Fairness, accountability, and transparency*, 90–99. 10.1145/3287560.3287563

[CR22] Green, B., & Chen, Y. (2019b). The principles and limits of algorithm-in-the-loop decision making. *ACM on Human-Computer Interaction, 3(CSCW)*. 10.1145/3359152

[CR23] Green, B., & Chen, Y. (2021). Algorithmic risk assessments can alter human decision-making processes in high-stakes Government Contexts. *ACM on Human–Computer Interaction, 5*(CSCW2). 10.1145/3479562

[CR24] Grgic-Hlaca N, Engel C, Gummadi KP (2019). Human decision making with machine assistance: An experiment on bailing and jailing. SSRN Electronic Journal.

[CR25] Lambe KA, O'Reilly G, Kelly BD, Curristan S (2016). Dual-process cognitive interventions to enhance diagnostic reasoning: A systematic review. BMJ Quality & Safety.

[CR26] Larson, J., Mattu, S., Kirchner, L., & Angwin, J. (2016). How we analyzed the COMPAS recidivism algorithm. *ProPublica*. https://www.propublica.org/article/how-we-analyzed-the-compas-recidivism-algorithm

[CR27] López-Ossorio JJ, González Álvarez JL, Andrés Pueyo A (2016). Eficacia predictiva de la valoración policial del riesgo de la violencia de género. Psychosocial Intervention.

[CR28] Lyell D, Coiera E (2017). Automation bias and verification complexity: A systematic review. Journal of the American Medical Informatics Association.

[CR29] Lyell, D., Magrabi, F., Raban, M. Z., Pont, L. G., Baysari, M. T., Day, R. O., & Coiera, E. (2017). Automation bias in electronic prescribing. *BMC Medical Informatics and Decision Making, 17*(1). 10.1186/S12911-017-0425-5 10.1186/s12911-017-0425-5 PMC5356416 28302112

[CR30] Marquardson J, Grimes M (2018). Supporting better decisions: How order effects influence decision support system alignment. Interacting with Computers.

[CR31] MartínezGaray L (2016). Errores conceptuales en la estimación de riesgo de reincidencia: La importancia de diferenciar sensibilidad y valor predictivo, y estimaciones de riesgo absolutas y relativas. Revista Española De Investigación Criminológica.

[CR32] Ministerio Público Fiscal de la Ciudad Autónoma de Buenos Aires. (2020). *Innovación e inteligencia artificial*. https://mpfciudad.gob.ar/institucional/2020-03-09-21-42-38-innovacion-e-inteligencia-artificial

[CR33] Ministerstwo Sprawiedliwości. (2021). *Algorytm SLPS*. https://www.gov.pl/web/sprawiedliwosc/algorytm

[CR34] Ministry of Justice. (2013). *Offender assessment system (OASys)*. Data.Gov.Uk. https://www.data.gov.uk/dataset/911acd3c-495f-48ca-88b6-024210868b06/offender-assessment-system-oasys

[CR35] Mosier, K. L., & Manzey, D. (2019). Humans and automated decision aids: A match made in heaven? *Human performance in automated and autonomous systems*, 19–42. 10.1201/9780429458330-2

[CR36] Niiler, E. (2019). Can AI Be a Fair Judge in Court? Estonia thinks so. *WIRED*. https://www.wired.com/story/can-ai-be-fair-judge-court-estonia-thinks-so/

[CR37] Obermeyer Z, Powers B, Vogeli C, Mullainathan S (2019). Dissecting racial bias in an algorithm used to manage the health of populations. Science.

[CR38] O’Neil, C. (2016). *Weapons of math destruction: How big data increases inequality and threatens democracy*. Crown Publishing Group.

[CR39] OpenAI. (2023). *ChatGPT* (Jun 22 version) [Large language model]. https://chat.openai.com/chat

[CR40] Parasuraman R, Mustapha M (1996). Automation and human performance.

[CR41] Ponce J (2022). Reserva de humanidad y supervisión humana de la Inteligencia artificial. El Cronista Del Estado Social y Democrático De Derecho.

[CR42] Portela, M., & Álvarez, T. (2022). *Towards a meaningful human oversight of automated decision-making systems*. Digital Future Society. https://digitalfuturesociety.com/report/towards-a-meaningful-human-oversight-of-automated-decision-making-systems/

[CR43] Portela, M., Castillo, C., Tolan, S., Karimi-Haghighi, M., & Pueyo, A. A. (2022). *A comparative user study of human predictions in algorithm-supported recidivism risk assessment*. arXiv. 10.48550/arxiv.2201.11080

[CR44] Raghu, M., Blumer, K., Corrado, G., Kleinberg, J., Obermeyer, Z., & Mullainathan, S. (2019). *The algorithmic automation problem: Prediction, triage, and human effort*. ArXiv. 10.48550/arXiv.1903.12220

[CR45] Rastogi C, Zhang Y, Wei D, Varshney KR, Dhurandhar A, Tomsett R (2022). Deciding fast and slow: The role of cognitive biases in AI-assisted decision-making. Proceedings of the ACM on Human–computer Interaction.

[CR46] Saura, G., & Aragó, L. (2021). Un algoritmo impreciso condiciona la libertad de los presos. *La Vanguardia*. https://www.lavanguardia.com/vida/20211206/7888727/algoritmo-sirve-denegar-permisos-presos-pese-fallos.html

[CR47] Skeem J, Scurich N, Monahan J (2020). Impact of risk assessment on judges’ fairness in sentencing relatively poor defendants. Law and Human Behavior.

[CR48] Solans, D., Beretta, A., Portela, M., Castillo, C., & Monreale, A. (2022). *Human response to an AI-based decision support system: A user study on the effects of accuracy and bias.* arXiv. 10.48550/arXiv.2203.15514

[CR49] Soler, C. (2013). *RisCanvi. Protocolo de evaluación y gestión del riesgo de violencia con población penitenciaria* [PowerPoint slides]. Slideplayer. https://slideplayer.es/slide/7242758/

[CR50] Tversky A, Kahneman D (1974). Judgment under uncertainty: Heuristics and biases: biases in judgments reveal some heuristics of thinking under uncertainty. Science.

[CR51] Valdivia, A., Hyde-Vaamonde, C., & García-Marcos, J. (2022). *Judging the algorithm: A case study on the risk assessment tool for gender-based violence implemented in the Basque country*. arXiv. 10.48550/arXiv.2203.03723

[CR52] Vicente L, Matute H (2023). Humans inherit artificial intelligence biases. Scientific Reports.

[CR53] Wagner B (2019). Liable, but not in control? Ensuring meaningful human agency in automated decision-making systems. Policy & Internet.

[CR54] Wei, J. (2019). China uses AI assistive tech on court trial for first time. *ChinaDaily*. https://www.chinadaily.com.cn/a/201901/24/WS5c4959f9a3106c65c34e64ea.html

